# Velvet Antler Methanol Extracts Ameliorate Parkinson's Disease by Inhibiting Oxidative Stress and Neuroinflammation: From *C. elegans* to Mice

**DOI:** 10.1155/2021/8864395

**Published:** 2021-01-08

**Authors:** Ying Liu, Hongyuan Li, Yunfei Li, Min Yang, Xiaohui Wang, Yinghua Peng

**Affiliations:** ^1^State Key Laboratory for Molecular Biology of Special Economic Animal, Institute of Special Animal and Plant Sciences, Chinese Academy of Agricultural Sciences, Changchun, Jilin 130112, China; ^2^Laboratory of Chemical Biology, Changchun Institute of Applied Chemistry, Chinese Academy of Sciences, Changchun, Jilin 130022, China; ^3^State Key Laboratory of Medicinal Chemical Biology, Nankai University, Tianjin 300071, China; ^4^Department of Pharmaceutical Engineering, College of Humanities & Information, Changchun University of Technology, Changchun 130122, China; ^5^Department of Applied Chemistry and Engineering, University of Science and Technology of China, Hefei 230026, China

## Abstract

Velvet antler is the traditional tonic food or medicine used in East Asia for treating aging-related diseases. Herein, we try to dissect the pharmacology of methanol extracts (MEs) of velvet antler on Parkinson's disease (PD). *Caenorhabditis elegans* studies showed that MEs decreased the aggregation of *α*-synuclein and protected oxidative stress-induced DAergic neuron degeneration. *In vitro* cellular data indicated that MEs suppressed the LPS-induced MAPKs and NF-*κ*B activation, therefore inhibiting overproduction of reactive oxygen species, nitric oxide, tumor necrosis factor-*α*, and interleukin-6; blocking microglia activation; and protecting DAergic neurons from the microglia-mediated neurotoxicity. *In vivo* MPTP-induced PD mouse investigations found that MEs prevented MPTP-induced neuron loss in the substantia nigra and improved the behavioral rotating rod performance in MPTP-treated mice by increasing the expression level of tyrosine hydroxylase (TH) and downregulating *α*-synuclein protein expression. In all, these results demonstrate that MEs ameliorate PD by inhibiting oxidative stress and neuroinflammation.

## 1. Introduction

Parkinson's disease (PD) is the second most common neurodegenerative disorder after Alzheimer's disease and is still incurable [1]. It is characterized by motor symptoms such as uncontrollable tremor, muscle stiffness, and slowness of movement [2]. PD is characterized by severe degeneration of dopaminergic (DAergic) neurons in substantia nigra (SN) and depletion of dopamine in the striatum. The etiology and pathogenesis of PD so far have not been completely elucidated, although current theories suggest that oxidative stress and neuroinflammation exert DAergic neuron demise and are involved in neuronal degeneration of PD [3].

Currently, there are few therapeutic options for preventing and treating PD, which only treat symptoms and do not retard DAergic neuron degeneration. Velvet antler has been used as traditional Chinese medicine and tonic food in East Asia for thousands of years [4], which has been reported to exert anti-inflammatory and antiaging effects [5, 6]. Methanol extracts (MEs) of velvet antler, which are rich in terpenoids, phenol, steroids, lipids, and glycosides and have no protein substances, protect against oxidative stress in *Caenorhabditis elegans* (*C. elegans*) [7]. Herein, we try to investigate whether MEs could ameliorate PD based on *C. elegans* and mouse models. MEs were found to inhibit reactive oxygen species (ROS) and neuroinflammation and therefore prevented the degenerations of DAergic neurons, which indicate that MEs would be an effective therapeutic agent against PD.

## 2. Materials and Methods

### 2.1. Materials

Velvet antler from sika deer (*Cervus nippon*) was provided by the Zuojia Sika Deer Farm (Jilin, China). The preparation and composition of velvet antler methanol extracts (MEs) were previously described [7]. Simply, 7 g of antler velvet powder was mixed with 210 mL methanol and was refluxed at 80°C for 1 h. The supernatant was obtained after centrifugation at 8000 g for 15 min. The methanol solvent was removed under vacuum with a rotary evaporator. The yield of MEs was 3.2% (*w*/*w*) of the dried sample. 6-Hydroxydopamine (6-OHDA), 2,3-diaminonaphthalene, 2′,7′-dichlorodihydrofluorescin diacetate (DCFH-DA), crystal violet, BCA assay kit, 1-methyl-4-phenyl-1,2,3,6-tetrahydropyridine (MPTP), and hematoxylin/eosin were obtained from Sigma-Aldrich (St. Louis, MO, USA). Interleukin-6 (IL-6) and tumor necrosis factor *α* (TNF-*α*) ELISA kits were purchased from Abcam (Cambridge, MA, USA). The Cell Death Detection kit was purchased from Roche Applied Science (Basel, Switzerland). The primary antibodies against IBA-1, p-ERK, p-JNK, p-p38, GAPDH, p-p65, p-Akt, tyrosine hydroxylase (TH), and *α*-synuclein as well as the horseradish peroxidase- (HRP-) conjugated secondary antibody were purchased from Cell Signaling Technology (Beverly, MA, USA). Alexa Fluor 568 to goat IgG secondary antibody was purchased from Thermo Fisher Scientific (Waltham, MA, USA).

### 2.2. *C. elegans* DAergic Degeneration Measurement

A transgenic strain BZ555, which expresses GFP in DAergic neurons through the dat-1::GFP reporter system, was used to assess the effect of MEs on DAergic neurons. Adult worms were incubated for 6 h at 20°C and allowed to lay eggs; then, synchronized worms at the L4 larval stage were treated with 6-OHDA. At the end of exposure, worms were spread on the NGM OP50 plates with or without MEs. Worms at days 1, 2, and 3 past the adult stage were mounted onto 2% agarose pads and immobilized with 2 mM levamisole and imaged with a Nikon TS2-FLfluorescence microscope. DAergic neurons were counted by inspecting the GFP fluorescence, which could be quantified with ImageJ. At least 30 worms were examined with three replicates.

### 2.3. *C. elegans* Basal Slowing Response (BSR) Assay

The *C. elegans* strain N2 was handled according to standard procedures and grown at 20°C. N2 adult worms were incubated for 6 h at 20°C and allowed to lay eggs. The synchronized worms at the L4 larval stage were treated with M9 buffer containing 10 mM ascorbic acid and 50 mM 6-OHDA for 1 h. The worms were then washed in M9 buffer and spread on NGM OP50 plates with or without MEs. Ten worms at days 1, 2, and 3 past the adult stage were collected by a washing plate with M9 buffer and then transferred to plates with or without a ring-shaped OP50 lawn. 5 min later, the number of body bending was counted to assess the locomotor rate in 20 s duration. Data is expressed as the difference (Δ) in body bending per 20 s between worms in OP50 seeded plates and plates without food, which is a measurement of 6-OHDA oxidation damage on dopaminergic neurons in *C. elegans*. At least three replicates were performed independently.

### 2.4. *C. elegans α*-Synuclein Aggregation Measurement

A transgenic strain, NL5901[unc-54p::alpha synuclein::YFP+unc-119(+)], which stably expresses human alpha synuclein protein tagged with yellow fluorescent protein, was used to assess the PD in the worm. Briefly, NL5901 strain nematodes were exposed to MEs from the L4 larval stage to days 3 and 5 past the adult stage. At the end of exposure, worms were mounted onto 2% agarose pads and immobilized with 2 mM levamisole. To monitor the *α*-synuclein aggregation, YFP protein was microscopically visualized and photographed using a Nikon TS2-FLfluorescence microscope. At least 30 worms examined with three replicates were imaged, and the fluorescence signals were quantified in each worm with ImageJ software.

### 2.5. Cell Viability Assay

A mouse microglial BV2 cell line was cultured in Dulbecco's modified Eagle's medium (DMEM) containing 10% FBS, 4 mM glutamine, 100 U/mL penicillin, and 100 *μ*g/mL streptomycin at 37°C in a 5% CO_2_ incubator. BV2 cells were exposed to different treatments as indicated. After 24 h treatment, cells were fixed with 3.7% paraformaldehyde for 5 min and then stained with 0.05% crystal violet for 15 min. The plates were subsequently washed with tap water and dried for 30 min at room temperature; 200 *μ*L of methanol was added to each well, and the plates were shaken for 15 min at room temperature to dissolve the dye. Absorbance at 540 nm was measured using a plate reader.

### 2.6. ROS Measurement in BV2 Cells

BV2 cells were exposed to different treatments for 24 h as indicated. Cells were washed with PBS and then loaded with H_2_DCFDA at a final concentration of 5 *μ*M in a serum-free medium. H_2_DCFDA is a nonfluorescent and cell-permeable probe that is converted into 2′,7′-dichlorodihydrofluorescein after intracellular deacetylation and is subsequently oxidized to highly fluorescent dichlorofluorescein (DCF). Cells were incubated for 15 min and then washed. Cellular fluorescence was viewed by a Nikon TS2-FLfluorescence microscope.

### 2.7. NO Assay

BV2 cells were exposed to different treatments for 24 h as indicated. 100 *μ*L of supernatant media was removed after cells were treated for 24 h and added to flat black 96-well microfluor plates (Thermo Scientific, Waltham, MA, USA). Subsequently, 10 *μ*L of 2,3-diaminonaphthalene (0.05 mg/mL in 0.62 M HCl) was added to each well and incubated for 15 min. The reaction was quenched by the addition of 5 *μ*L of 3 M NaOH, and the plate was read on a plate reader with excitation at 360 nm and emission at 430 nm.

### 2.8. TNF-*α* and IL-6 ELISA

BV2 cells were exposed to different treatments as indicated. After 24 h treatment, culture supernatants were collected for TNF-*α* and IL-6 ELISAs, which were performed according to the manufacturer's instructions (Abcam, Cambridge, MA, USA). Briefly, the capture antibody was added in each well of a 96-well ELISA plate and incubated overnight at 4°C. The plate was washed with phosphate-buffered saline (PBS) with 0.05% Tween 20 solution (PBST) five times and then incubated with 100 *μ*L supernatant/each well for 2 h at room temperature. Following five PBST washings, biotin-conjugated antibody was added to each well and incubated for 1 h at room temperature. After five PBST washings, diluted avidin-HRP was added and the plate was incubated at room temperature for 30 min. After washing the plate five times, tetramethylbenzidine (TMB) substrate was added to each well, and the color was developed in the dark for 10-30 min at room temperature. The color reaction was stopped by adding 1 M H_3_PO_4_. The absorbance at 450 nm was measured on a plate reader, and 620 nm was chosen as the reference wavelength. The concentration of TNF-*α* and IL-6 was calculated using a mouse TNF-*α* and IL-6 standard working curve, respectively.

### 2.9. Iba-1 Immunofluorescence Staining

BV2 cells were exposed to different treatments as indicated. After 24 h treatment, cells were fixed with 4% paraformaldehyde. 1% bovine serum albumin (BSA) in PBS was used for blocking for 30 min. The cells were incubated overnight at 4°C with Iba-1 primary antibody diluted 1 : 250 in 1% BSA in PBS. After five washings with PBS, the cells were incubated with a secondary Alexa Fluor 568-conjugated anti-goat IgG for 1 h at room temperature. The cellular nucleus was stained with 1 *μ*g/mL of Hoechst 33258 for 5 min. The staining of BV2 cells was visualized by a Nikon TS2-FLfluorescence microscope.

### 2.10. TUNEL Assay

BV2 cells were exposed to different treatments as indicated. After 24 h treatment, cells were fixed with 4% paraformaldehyde, and terminal deoxynucleotidyl transferase dUTP nick end labeling (TUNEL) staining was performed by the Cell Death Detection kit (Roche Applied Science, Basel, Switzerland) according to the manufacturer's instruction. DNA fragmentation was detected using a Nikon TS2-FLfluorescence microscope.

### 2.11. Western Blotting

The lysate samples were first resolved in 12% sodium dodecyl sulfate-polyacrylamide gel electrophoresis (SDS-PAGE) and then transferred to nitrocellulose membranes, followed by blocking membranes with 5% nonfat dry milk in TBST buffer (25 mM Tris-HCl, 140 mM NaCl, 0.05% Tween 20, pH 7.5) for 1 h and incubation with the appropriate primary antibody (0.5 *μ*g/mL) at 4°C overnight. After being washed five times by TBST, the membrane was incubated with horseradish peroxidase-conjugated secondary antibody (50 ng/mL) for 1 h at room temperature. After sufficient washing, antibody complexes attached to the membrane were visualized by a Tanon-5200 Multi when reacting with SuperSignal West Pico Chemiluminescent Substrate (Pierce, Rockford, IL, USA). ImageJ was used for densitometric analysis.

### 2.12. *In Vivo* Mouse Behavioral Studies

Pathogen-free adult male C57BL/6 mice weighing 20–25 g were used in all experiments (Liaoning Changsheng Biotechnology, China). Mice were housed in temperature-controlled (18–21°C) and light-controlled (12 h light-dark cycle; lights on at 7:00 am) rooms with standard rodent food and water available ad libitum and allowed to habituate to the holding facility for ≥1 week prior to experimentation. All the animal-handling procedures were performed in strict accordance with the regulations for the Administration of Affairs Concerning Experimental Animals approved by the State Council of the People's Republic of China (11-14-1988).

The mice that pretrained on the rotating rod and had no difference in the time of staying on the rod were randomly divided into three groups (6 per group): the control group (intraperitoneal injection of a saline solution for 5 days), MPTP-treated groups (intraperitoneal injection of 30 mg/kg MPTP for 5 days), and MPTP plus ME-treated group (intraperitoneal injection of 30 mg/kg MPTP and 30 mg/kg MEs for 5 days). The animals were subjected to behavioral testing 48 h after the final injection and then sacrificed.

PD-induced behavioral changes were measured by the rotarod performance test as described previously [8]. In brief, mice performed three 10 min pretraining trials daily for 3 consecutive days before MPTP treatment. The rotarod performance test used a rod with a diameter of 3 cm and set at 40 rpm rotation. Mice were placed on the rotating rod, and the time for which each mouse maintained balance on the rod was recorded. Each mouse was tested three times with an intertrial interval of 20 min. The average time was considered the final score.

### 2.13. Immunohistochemistry

After the behavioral studies, brain tissues were dissected and fixed in universal tissue-fixed fluid at 4°C for 24 and then embedded in paraffin. Immunohistochemistry (IHC) was then performed. The tissue slides (3 *μ*m) were deparaffinized with xylene and rehydrated in alcohol, washed with PBS, and incubated in 10 mM citrate buffer (pH 7.4) at 90°C for 15 min. The slides were then treated with 0.3% hydrogen peroxide in methanol at 4°C for 30 min to inactivate endogenous peroxidases. After blocking with 5% BSA for 25 min at 25°C, the tissues were washed with PBS and incubated with primary antibody overnight at 4°C. Subsequently, they were incubated with secondary antibody for 30 min. DAB was used as the chromogenic agent, and hematoxylin was used as the dye reagent. The tissue slides were blocked and then observed under a microscope.

### 2.14. Immunofluorescence Staining of NF-*κ*B in Substantia Nigra (SN)

After the behavioral studies, brain tissues were dissected and fixed in 4% paraformaldehyde and embedded in paraffin. The brains were then cut into 3 *μ*m coronal sections with a paraffin microtome and followed by heat-induced antigen retrieval. Sections containing substantia nigra regions were subjected to immunostaining. Endogenous peroxidase activity was quenched by incubation in 1% hydrogen peroxide in methanol for 30 min and then cleared in PBS for 5 min. The sections were blocked for 30 min with bovine serum albumin diluted in PBS. These sections were incubated with primary antibody against NF-*κ*B protein (ab16502, Abcam, UK) overnight at 4°C. After washing in PBS, the sections were incubated in cy3-conjugated secondary antibody (Servicebio, China) for 1 h at room temperature. Washing of the sections was again done three times and then incubated with DAPI solution at room temperature for 10 min. The sections were subsequently washed with PBS and viewed under a fluorescence microscope.

### 2.15. Hematoxylin/Eosin Staining

Tissues were fixed in universal tissue fixation fluid at 4°C for 24 h and embedded in paraffin. Tissue sections (3 *μ*m) were deparaffinized and stained with hematoxylin/eosin (H-E). At least five paraffin sections from each tissue were used for H-E staining.

## 3. Results and Discussions

### 3.1. MEs Inhibit *α*-Synuclein Aggregation and Protect DAergic Neurons in *C. elegans*

**Figure 1:**
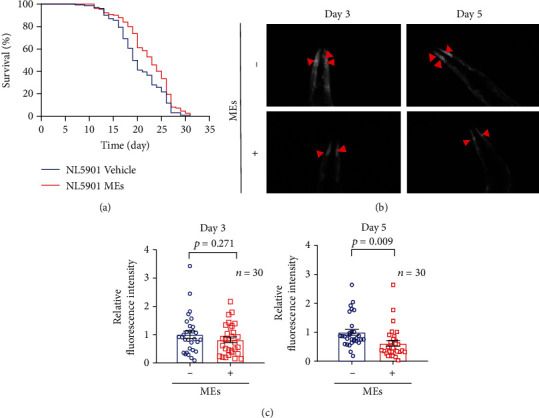
MEs decreased the aggregation and toxicity of *α*-synuclein in NL5901 worms. (a) The survival curve of NL5901 worms treated with MEs. (b) The endpoint microscope fluorescence image of *α*-synuclein aggregates in NL5901 worms treated with or without MEs on days 3 and 5 past the adult stage. (c) The quantitative analysis of the *α*-synuclein aggregates in NL5901 worms shown in (b). All the worms in the experiment were synchronized to the young adult stage and subsequently started to be exposed to 100 *μ*g/mL MEs. Error bars represented the SEM of three independent replicates of total worms.

*C. elegans* is a good model organism for the study of pathogenesis and drug discovery for PD [9], owing to several advantages including a completely sequenced genome [10], numerous mutants and multicolor reporter constructs freely available [11, 12], a rapid replication cycle, and ease of growing and maintenance as well as manipulation [13]. Increasing evidence suggests that oxidative stress plays a major role in the development of PD [14]. Our recent work showed that MEs of velvet antler protected against oxidative stress in *C. elegans*. Therefore, whether MEs could affect the progression of PD was investigated. NL5901 strain, which has been created by inserting human *α*-synuclein gene with YFP fusion construct driven by the unc-54 promoter, was used as a PD model. As shown in Figure 1(a), ME treatment significantly prolonged the lifespan of NL5901 worms when compared to the untreated control. NL5901 worms exhibited aggregation of *α*-synuclein (Figure 1(b)). The YFP intensities were quantitatively analyzed, and the mean fluorescent intensity in the control group was set as 1. Although not statistically significant, there was a trend which suggested that ME treatment decreased the *α*-synuclein-YFP intensities on day 3 (Figure 1(c)). Moreover, ME treatment significantly decreased the *α*-synuclein-YFP intensities on day 5 when compared to the untreated control (Figure 1(c)). These results indicate that MEs decrease the aggregation of *α*-synuclein and therefore improve the lifespan of the worm model of PD.

**Figure 2:**
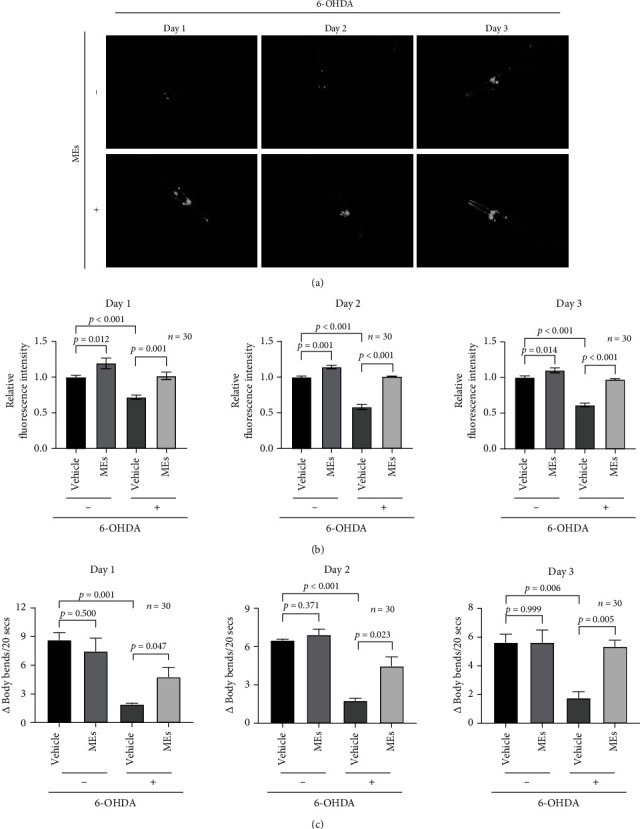
MEs protected against neuron injury induced by 6-OHDA in *C. elegans.* (a) The endpoint microscope fluorescence image of DAergic neurons in BZ555 worms treated with or without MEs on days 1, 2, and 3 past the adult stage. The BZ555 worms synchronized at the L4 larval stage were exposed to 6-OHDA and subsequently started to be exposed to 100 *μ*g/mL MEs for 1-3 days. (b) The quantitative analysis of the intensity of DAergic neurons shown in (a). (c) The difference of the average number of body bends per 20 s between N2 worms in OP50 seeded plates and plates without food. N2 worms synchronized at the L4 larval stage were exposed to 6-OHDA and subsequently started to be exposed to 100 *μ*g/mL MEs for 1-3 days. Data were expressed as the mean ± SEM.

PD is typically associated with degeneration of the DAergic neurons [15], which are particularly prone to oxidative stress [16]. Whether MEs could protect DAergic neurons was next investigated in *C. elegans*. In contrast to rodents which have 10,000–20,000 DAergic neurons, or humans which have greater than 40,000 DAergic neurons [17], *C. elegans* have only eight DAergic neurons: two anterior, four cephalic, and two posterior deirid neurons [18], which makes the *in situ* investigation of the vulnerability of DAergic neurons to oxidative stress possible. BZ555 strain, in which all DAergic neurons are tagged with GFP by fusing with DAT-1, was used to visualize the bodies of DAergic neurons. 6-Hydroxydopamine (6-OHDA), a redox cycling dopamine analog and an oxidative neurotoxin, was used to lesion dopaminergic pathways and generate an experimental model for PD [19]. BZ555 worms were treated as indicated, and the green fluorescence was analyzed in the nerve ring (Figure 2(a) and Figure S1), which contains GFP-tagged DAergic neurons. Compared to the untreated control, 6-OHDA treatment caused a significant shrinkage of the soma of DAergic neurons (Figure 2(b)). ME treatment slightly increased the nerve ring of DAergic neurons under the normal condition and significantly ameliorated the 6-OHDA-induced degeneration of DAergic neurons (Figure 2(b)).

To confirm the results from the transgene BZ555 worms, the effect of MEs on the functionality of DAergic neurons under the oxidative stress was evaluated by the basal slowing response (BSR) assay in the wild-type N2 worms. This behavioral assay measures the ability of wild worms to slow down their rate of locomotion when they encounter a bacterial lawn, which is mediated by DA. As shown in Figure 2(c), 6-OHDA treatment induced a reduction in BSR as compared to the untreated control. MEs increased the BSR value of N2 worms treated with 6-OHDA but did not affect the BSR of the wild-type worms under the normal condition (Figure 2(c)). These functional data revealed by BSR of the wild-type N2 worms are consistent with the direct DAergic neuron observations in the transgene BZ555 worms. Together, these C. elegans data demonstrate that MEs inhibit *α*-synuclein aggregation and protect DAergic neurons in *C. elegans* from degeneration.

### 3.2. MEs Inhibit ROS and Proinflammatory Factors in the Activated Microglia

**Figure 3:**
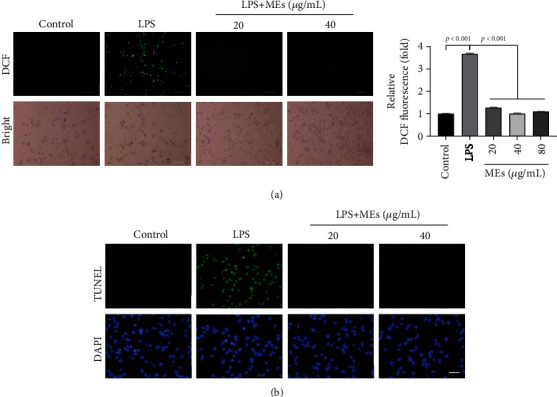
MEs inhibit ROS generation in the activated microglia. (a) The endpoint microscope fluorescence image of intracellular ROS of BV2 cells. Intracellular ROS generation was measured by fluorescence staining with H_2_DCF-DA. LPS (200 ng/mL)-activated BV2 cells were treated with MEs (20 and 40 *μ*g/mL), scale bar = 100 *μ*m. The quantitative analysis of the fluorescence intensity of DCF shown in (a). (b) TUNEL staining. LPS (200 ng/mL)-activated BV2 cells were treated with MEs (20 and 40 *μ*g/mL) for 24 h. DNA fragmentation was detected by TUNEL assay. Scale bar = 100 *μ*m.

The above *C. elegans* data implies that MEs of velvet antler inhibit the development of PD. To test whether MEs could be a potential means for preventing and treating PD, the effects of MEs were further tested in the mammalian system. Before moving to *in vivo* mouse studies, the actions of MEs were tested in the microglia, which are the main immune effector cells in the central nervous system (CNS) and play a major role in PD pathology [20]. Although a variety of potential sources for ROS exist in the CNS, the microglia generate large quantities of these reactive species [21]. Microglia BV2 cells were used herein since they reproduce many of the responses of primary microglia with high fidelity [22]. To explore the effect of MEs on the LPS-induced ROS production, the bacterial endotoxin, lipopolysaccharide (LPS), which has been the most extensively utilized microglia activator for the induction of DAergic neurodegeneration [23], was used to activate BV2 cells. Intracellular ROS generation was measured by fluorescence staining with H_2_DCF-DA. No apparent green fluorescence was observed for the untreated BV2 cells (Figure 3(a)). LPS stimulation caused the ROS burst in BV2 as revealed by the strong green fluorescence from DCF (Figure 3(a)), suggesting the activation of microglia. MEs significantly inhibited the LPS-induced DCF fluorescence intensity (Figure 3(a)), which indicates that MEs reduce the oxidative stress in the activated microglia. Excess ROS directly inflicts DNA damage, and TUNEL staining was performed to assess oxidative stress by measuring DNA fragmentation. Compared to the untreated control, LPS stimulation caused DNA fragmentation in BV2 cells as reflected by TUNEL fluorescence (Figure 3(b)). MEs protected against the LPS-induced DNA fragmentation (Figure 3(b)). Together, these results from microglia are consistent with the previous observation that MEs protect against oxidative stress in *C. elegans* [7]. It should be noted that the effect of MEs on cellular viability was measured by crystal violet staining. No apparent cellular toxicity was observed (Figure S2), even at the concentration of 80 *μ*g/mL of MEs, which eliminates the possibility that the observed ROS inhibition by MEs was due to the artifact like cell death.

**Figure 4:**
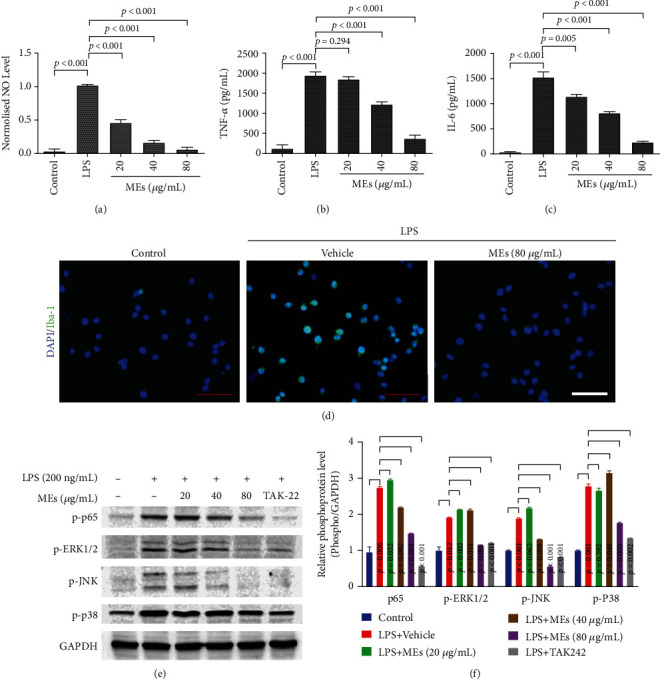
MEs inhibit proinflammatory factors by suppressing MAPKs and NF-*κ*B activation in the activated microglia. (a–c) MEs inhibited LPS-induced NO (a), TNF-*α* (b), and IL-6 (c). BV2 cells were treated with LPS (200 ng/mL) and indicated concentrations of MEs. After 24 h treatment, NO, TNF-*α*, and IL-6 in the supernatant were measured; data are expressed as mean ± SD. (d) The endpoint microscope immunofluorescence image of Iba-1 in BV2 cells. BV2 cells were treated with LPS (200 ng/mL) and indicated concentration of MEs for 24 h. Iba-1 immunofluorescence staining was subsequently performed. Scale bar = 100 *μ*m. (e, f) Effect of MEs on the LPS-induced phosphorylation of p65, ERK, JNK, and p38. BV2 cells were treated with LPS (200 ng/mL) and indicated concentrations of MEs for 1.5 h. Cell lysates were prepared, and the protein samples were analyzed by western blot analysis. TAK-242 (1 *μ*M), a classic TLR4 antagonist, was used as the control. Results are representative of those obtained from three independent experiments.

In addition to ROS, accumulating evidence points to activated microglia as a main source of several proinflammatory factors, including nitric oxide (NO), tumor necrosis factor-*α* (TNF-*α*), and interleukin-6 (IL-6), driving progressive DAergic neurodegeneration and PD development [24, 25]. The effect of MEs on the proinflammatory mediators in the activated microglia was investigated. LPS induced NO (Figure 4(a)), TNF-*α* protein (Figure 4(b)), and IL-6 protein (Figure 4(c)) overproduction in BV2 cells. MEs inhibited the LPS-induced NO (Figure 4(a)), TNF-*α* (Figure 4(b)), and IL-6 (Figure 4(c)) in a concentration-dependent manner. These results imply that MEs have antineuroinflammation activity.

Ionized calcium-binding adaptor molecule 1 (Iba-1) is specifically expressed in microglia/macrophages and is involved with the membrane ruffling and phagocytosis in activated microglia [26]. Iba-1 is upregulated during the activation of microglia, which is therefore used as the marker of microglia activation [27]. As shown in Figure 4(d), Iba-1 was expressed on the plasma of BV2 cells, where a green fluorescence signal was observed. LPS stimulation increased the expression of Iba-1, which was inhibited by MEs. Together, these data show that MEs inhibit microglial activation, which is consistent with the suppression of LPS-induced ROS and proinflammatory factor (NO, TNF-*α*, and IL-6) overproduction by MEs.

Oxidative stress and neuroinflammation cause neuronal cell degeneration [25, 28–31]. To directly explore whether MEs could protect neuron cells from the damages caused by oxidative stress and neuroinflammation from the activated microglia, the effect of conditional media from BV2 cells on SH-SY5Y cellular viability was investigated. SH-SY5Y cells were used as an *in vitro* model of DAergic neurons for PD research, because they possess many characteristics of DAergic neurons [32]. The conditioned medium from the LPS-treated BV2 cells caused an ~40% decrease of SH-SY5Y cellular viability (Figure S3). MEs decreased the cellular toxicity of conditioned media from the LPS-treated BV2 cells in a concentration-dependent manner (Figure S3). These results show that MEs could protect DAergic neurons from microglia-mediated neurotoxicity.

The expression of LPS-induced ROS and proinflammatory factors is governed by the MAPKs and NF-*κ*B [33, 34]. In order to dissect how MEs downregulate the LPS-induced ROS and proinflammatory factors, the effect of MEs on MAPKs and NF-*κ*B activities was measured. LPS stimulation significantly increased the phosphorylation of p65 subunit of NF-*κ*B, ERK1/2, JNK, and p38 (Figures 4(e) and 4(f)). TAK242, a classic TLR4 antagonist, was used here as a positive control. Similar to TAK242, MEs inhibited the LPS-induced phosphorylation of NF-*κ*B p65, ERK1/2, p38, and JNK in a concentration-dependent manner (Figures 4(e) and 4(f)).

Taken together, these results suggested that MEs suppressed the LPS-induced MAPK and NF-*κ*B activation, therefore inhibiting ROS and proinflammatory factors in the activated microglia and protecting DAergic neurons from the microglia-mediated neurotoxicity.

### 3.3. MEs Improve Parkinsonism in MPTP-Treated Mice

**Figure 5:**
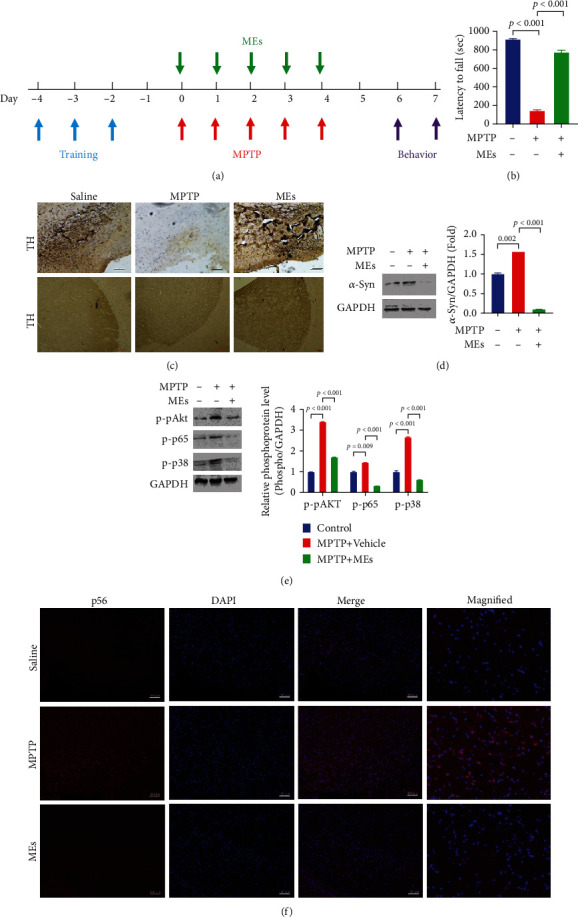
MEs improve parkinsonism in MPTP-treated mice. (a) Experimental timeline for the construction of the MPTP-induced PD mouse model and the administration of MEs. C57BL/6 mice (male, 7-8-week-old) were injected MPTP intraperitoneally at 30 mg/kg/day for five days, and MEs (30 mg/kg/day) were injected intraperitoneally for 5 days since the administration of MPTP. On day 6, the rotarod test was performed. On day 7, mice were sacrificed and tissues were prepared for immunohistochemical (IHC) and western blotting. (b) Rotarod behavioral performance of MPTP-induced PD mice after ME treatment. Data are presented as the mean ± SD (*n* = 6). (c) Immunohistochemistry for tyrosine hydroxylase (TH) in the substantia nigra (scale bar = 100 *μ*m) and striatum (scale bar = 1000 *μ*m). (d) Expression of *α*-syn by western blot analysis. The blots were reprobed to detect GAPDH as the internal control. (e) Effect of MEs on the LPS-induced phosphorylation of Akt, p65, and p38 in the striatum. The protein in obtained tissue was analyzed and quantified by western blotting. (f) Effect of MEs on the nuclear translocation of NF-*κ*B in substantia nigra (scale bar = 100 *μ*m). C57BL/6 mice (male, 7-8-week-old) were injected MPTP intraperitoneally at 30 mg/kg/day for five days, and MEs (30 mg/kg) were injected intraperitoneally for 5 days since the administration of MPTP. On day 7, mice were sacrificed and substantia nigra tissues were collected, and immunofluorescence was performed.

The etiology of PD indicates that ROS and proinflammatory factors [14, 28], particularly the generation of ROS, NO, and proinflammatory cytokines by activated microglia [35], mediates the majority of DAergic neuron destruction [20]. Cellular data shows that MEs of velvet antler inhibit ROS and neuroinflammation in the activated microglia and protect DAergic neurons from the microglia-mediated neurotoxicity. To confirm whether MEs could prevent and treat PD, the effect of MEs on the MPTP-induced PD mouse model was investigated. It should be noted that MPTP is the gold standard for toxin-based PD animal models [30, 31, 36], as it recapitulates the primary pathological and biochemical features of PD [37]. As shown in Figure 5(a), PD syndrome was induced by intraperitoneal administrations of MPTP, and MEs were administered once per day since the first MPTP injection for 5 days. Rotarod tests were performed 48 h after the final injection to determine whether MEs protected against the motor deficits caused by MPTP neurotoxicity. Compared to the vehicle control (918 s ± 12 s), the staying time of MPTP-treated mice on the rotating rod (145 s ± 16 s) was much shorter (Figure 5(b)). This is not surprising since subacute treatment of MPTP induces significant loss of neurons in the nigrostriatal pathway (Figure S4), which plays important roles in motor function [38]. ME treatment increased neurons in the substantia nigra of MPTP-treated mice (Figure S4) and substantially improved the performance of MPTP-treated mice to 776 s ± 29 s (Figure 5(b)). These results suggest that MEs improve the behavioral deficits in MPTP-induced PD mice.

PD is characterized by the degeneration of DAergic neuron in substantia nigra (SN), leading to a reduction of striatal dopamine [39]. Tyrosine hydroxylase (TH) catalyses the formation of L-dihydroxyphenylalanine (L-DOPA) [40], the rate-limiting step in the biosynthesis of dopamine [41]. Therefore, the TH level is closely associated with DAergic neuron function, and the reduction of the TH level in the brain tissues is a direct indication of DAergic neuron loss [42]. As shown in Figure 5(c), IHC analysis showed that MPTP-treated mice had decreased TH-positive neurons in the SN and striatum compared to the vehicle control group. MEs significantly attenuated the MPTP-induced decrease of TH-positive neurons in the SN and striatum (Figure 5(c)). These results indicated that MEs could significantly reverse the decrease of TH expression and the loss of DAergic neurons in the MPTP-induced PD mice, which is consistent with the notion that increasing nigrostriatal TH expression is an effective therapeutic strategy for PD [43].

*α*-Synuclein, a key protein critically involved in PD pathogenesis [44], is a major component of Lewy bodies [45], which are the neuropathological hallmarks of PD [46]. Aggregated *α*-synuclein interacts with the cell membrane of neurons to form pore-like structures and depolarize the membrane potential, which disrupts the normal functions of the cell and leads to DAergic neuron death and progress of PD [47, 48]. Therefore, *α*-synuclein is a therapeutic target for PD [49]. As shown in Figure 5(d), MPTP administration induced a significant upregulation of *α*-synuclein protein in the striatum compared to the vehicle control, which is in agreement with the observed decrease of staying time of MPTP-treated mice on the rotating rod. Much less *α*-synuclein protein expression was observed in the striatum of mice cotreated with MPTP and MEs (Figure 5(d)). These results indicate that MEs can protect PD development through downregulating *α*-synuclein protein expression.

Akt, NF-*κ*B, and p38 are dysregulated in the brain of PD patients [24, 50–52] and are the main components in the signal transduction pathway predominantly responsible for the generation of ROS and proinflammatory factors [53]. To investigate how MEs affect ROS and neuroinflammation in MPTP-induced PD mice, the effect of MEs on the phosphorylation of Akt, NF-*κ*B, and p38 in the striatum was measured. As shown in Figure 5(e), MPTP treatment induced a significant upregulation of p-Akt, p-p65, and p-p38 in the striatum when compared to the vehicle control group. MEs substantially reduced the MPTP-induced Akt, NF-*κ*B, and p38 activation (Figure 5(e)). In addition, NF-*κ*B nuclear translocation was found in MPTP-induced PD mice [30]; ME treatment could inhibit this nuclear translocation of NF-*κ*B in our study **(**Figure 5(f)**)**. In agreement with *in vitro* study, these *in vivo* results imply that MEs inhibit Akt, NF-*κ*B, and p38.

**Figure 6:**
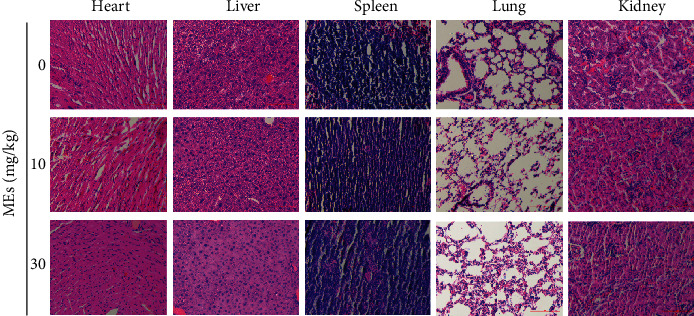
Acute toxicity of MEs in different organs. C57BL/6 mice (male, 7-8-week-old) were treated with indicated concentrations of MEs for 7 days. Mice were sacrificed, and different organs (heart, liver, spleen, lung, and kidney) were collected for hematoxylin and eosin (H&E) staining. Scale bar = 100 *μ*m.

Last, the acute toxicology of MEs was investigated. Hematoxylin and eosin- (H&E-) staining of different organs (including the heart, liver, kidney, spleen, and lung) of mice treated with MEs for 7 days was examined (Figure 6). No apparent damage was detected in the examined organs, which indicates low safety concern of MEs. This is not surprising since velvet antler has been used as medicine and tonic food in East Asia for over two thousand years [4].

## 4. Conclusions

In all, this study dissected the pharmacology of MEs of velvet antler in PD. The *C. elegans* PD model studies show that MEs inhibit *α*-synuclein aggregation and protect DAergic neurons from degeneration. *In vitro* microglia cellular data indicate that MEs suppressed the LPS-induced MAPK and NF-*κ*B activation, therefore inhibiting ROS and proinflammatory factors as well as the activation of microglia and protecting DAergic neurons from the microglia-mediated neurotoxicity. *In vivo* MPTP-induced PD mouse investigations demonstrate that MEs prevent MPTP-induced neuron loss in the substantia nigra and improve parkinsonism in MPTP-treated mice by increasing the expression level of TH and downregulating *α*-synuclein protein expression. Since MEs of velvet antler have no apparent toxicology, MEs would have good translational potential for preventing and treating PD.

## Data Availability

The accessibility data used to support the findings of this study were collected according to scientific research criteria and can be available from the corresponding authors upon request.

## Supplementary Materials

Supplementary materials Figure S1: MEs had little damage to the DAergic neurons. The endpoint microscope fluorescence image of DAergic neurons in BZ555 worms treated with or without MEs o n days 1, 2, and 3 past the adult stage. The BZ555 worms synchronized at theL4 larval stage were not exposed to 6-OHDA and subsequently started to be exposed to 100 *μ*g/mL MEs for 1-3 days. Figure S2: MEs had little cellular toxicity. BV2 cells were treated with different concentrations of MEs for 24 h. Cellular viability was measured by MTT assay. Data are expressed as mean ± SD, n.s., *p* > 0.05. Figure S3: MEs protect DAergic neurons from microglia-mediated neurotoxicity. BV2 cells were treated with LPS (200 ng/mL) and indicated concentrations of MEs for 30 h. The conditioned medium of microglial cells was then transferred to SH-SY5Y cell culture. After 24 h of incubation, the cell viability of SH-SY5Y cells was analyzed by MTT assay. Data are expressed as the mean ± SD. Figure S4: ME treatment increased neurons in the substantia nigra of MPTP-treated mice. C57BL/6 mice (male, 7-8-week-old) were injected MPTP intraperitoneally at 30 mg/kg/day for five days, and MEs (30 mg/kg) were injected intraperitoneally for 5 days since the administration of MPTP. On day 7, mice were sacrificed, and substantia nigra tissues were collected, and hematoxylin and eosin (H&E) staining was performed. Scale bar = 100 *μ*m, right part (40x magnification).
