# Supplementary material for: Velvet Antler Methanol Extracts Ameliorate Parkinson's Disease by Inhibiting Oxidative Stress and Neuroinflammation: From C. elegans to Mice
Source: Oxid Med Cell Longev. 2021 Jan 8;2021:8864395. doi: 10.1155/2021/8864395 (PMC7811427; doi:10.1155/2021/8864395)
Supplement: Supplementary materials — Figure S1: MEs had little damage to the DAergic neurons. The endpoint microscope fluorescence image of DAergic neurons in BZ555 worms treated with or without MEs o n days 1, 2, and 3 past the adult stage. The BZ555 worms synchronized at theL4 larval stage were not exposed to 6-OHDA and subsequently started to be exposed to 100 μg/mL MEs for 1-3 days. Figure S2: MEs had little cellular toxicity. BV2 cells were treated with different concentrations of MEs for 24 h. Cellular viability was measured by MTT assay. Data are expressed as mean ± SD, n.s., p > 0.05. Figure S3: MEs protect DAergic neurons from microglia-mediated neurotoxicity. BV2 cells were treated with LPS (200 ng/mL) and indicated concentrations of MEs for 30 h. The conditioned medium of microglial cells was then transferred to SH-SY5Y cell culture. After 24 h of incubation, the cell viability of SH-SY5Y cells was analyzed by MTT assay. Data are expressed as the mean ± SD. Figure S4: ME treatment increased neurons in the substantia nigra of MPTP-treated mice. C57BL/6 mice (male, 7-8-week-old) were injected MPTP intraperitoneally at 30 mg/kg/day for five days, and MEs (30 mg/kg) were injected intraperitoneally for 5 days since the administration of MPTP. On day 7, mice were sacrificed, and substantia nigra tissues were collected, and hematoxylin and eosin (H&E) staining was performed. Scale bar = 100 μm, right part (40x magnification). [file 8864395.f1.docx]

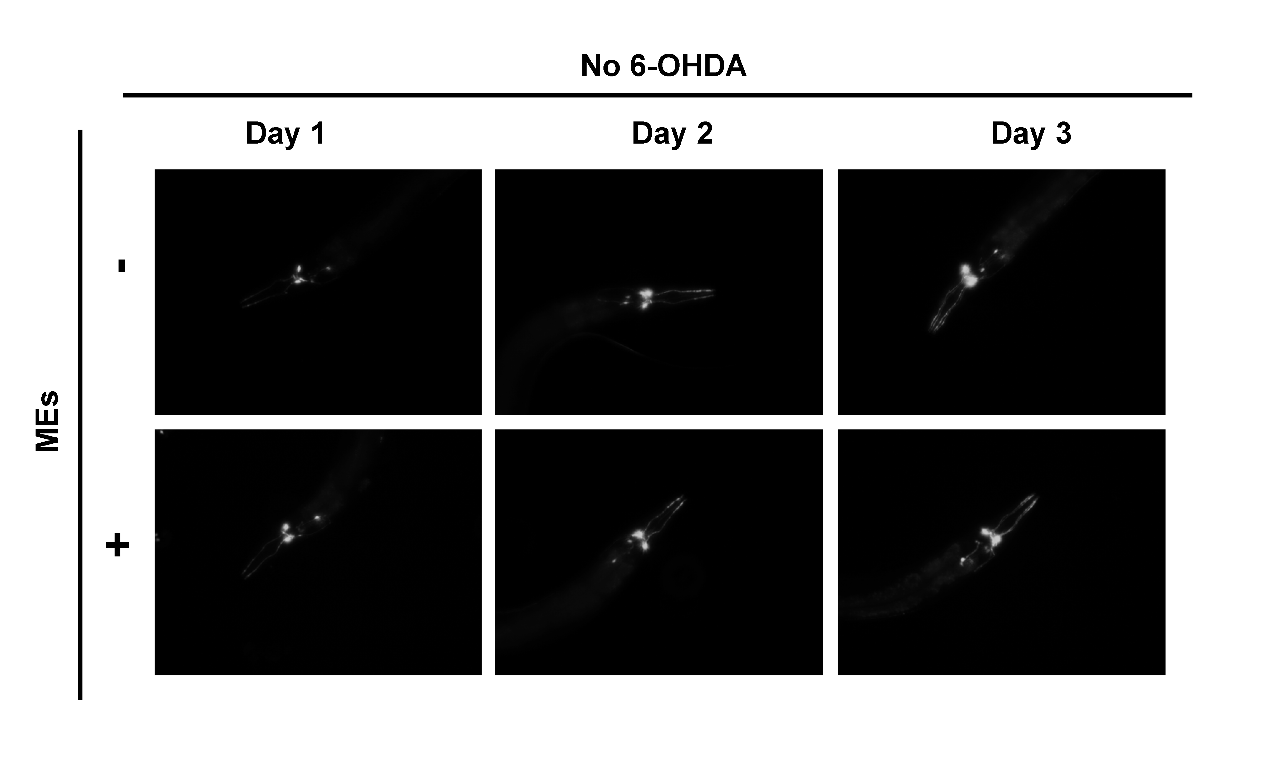


**Figure S1. MEs had little damage to the DAergic neurons.** The endpoint microscope fluorescence image of DAergic neurons in BZ555 worms treated with or without MEs at day 1, 2, 3 past adult stage. The BZ555 worms synchronized at L4 larval stage were not expose to 6-OHDA, and subsequently started to expose to 100 μg/mL MEs for 1-3 days.


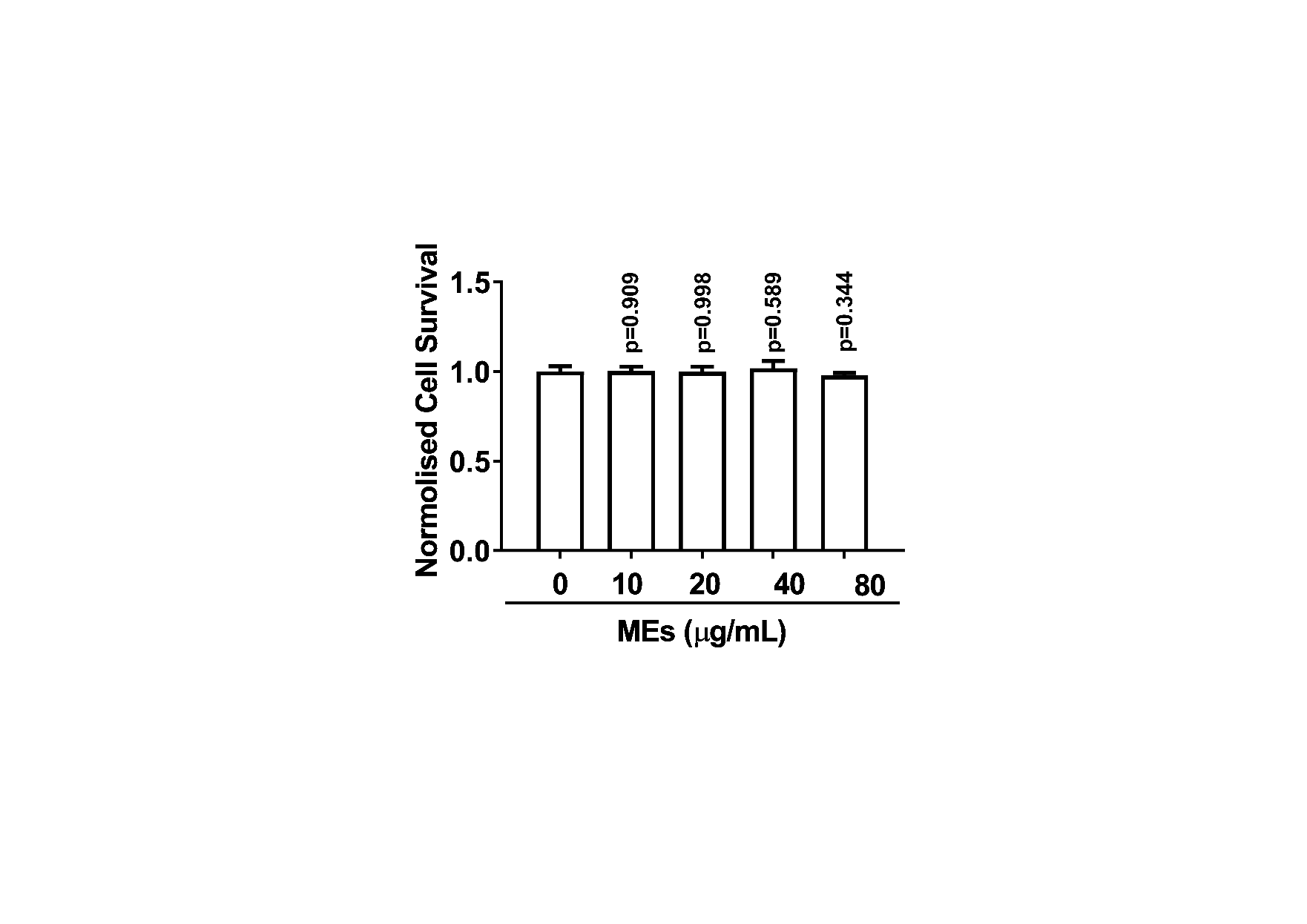


**Figure S2. MEs had little cellular toxicity.** BV-2 cells were treated with different concentrations of MEs for 24 h. Cellular viability was measured by MTT assay. Data are expressed as mean ± SD.


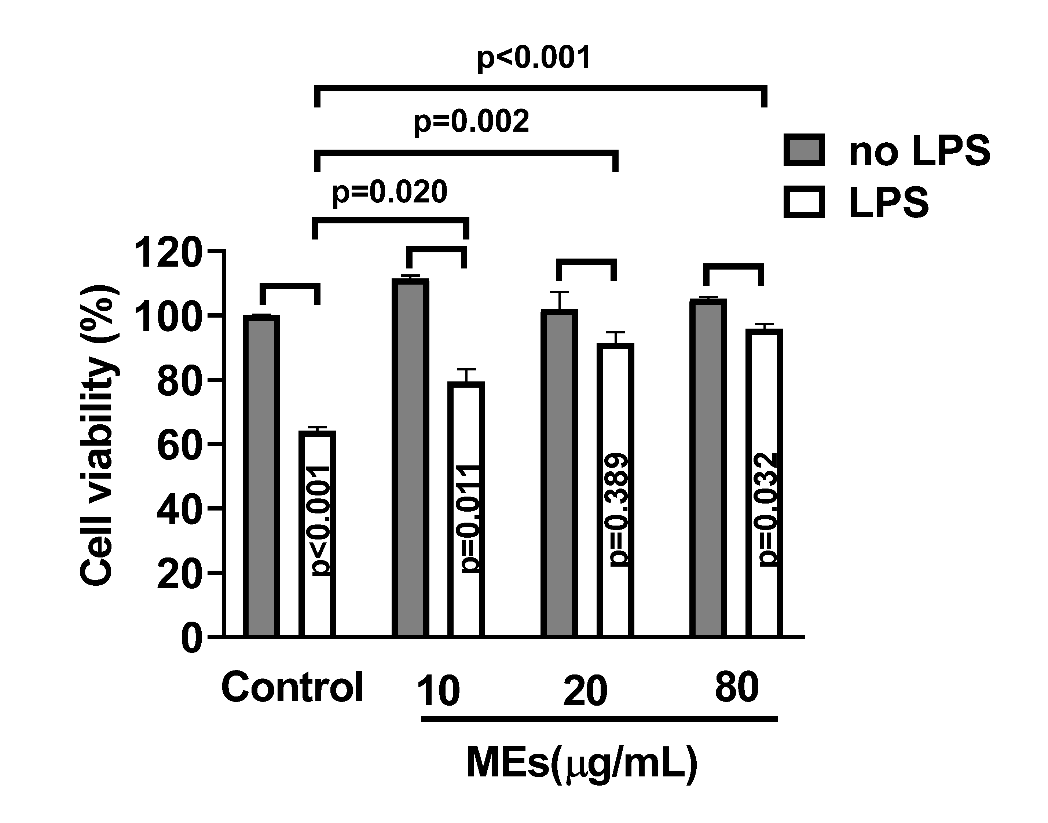


**Figure S3.** **MEs protect DAergic neurons from the microglia-mediated neurotoxicity.** BV-2 cells were treated with LPS (200 ng/mL) and indicated concentrations of MEs for 30 h. The conditioned medium of microglial cells was then transferred to SH-SY5Y cell culture. After 24h of incubation, the cell viability of SH-SY5Y cells was analyzed by MTT assay. Data are expressed as mean ± SD.


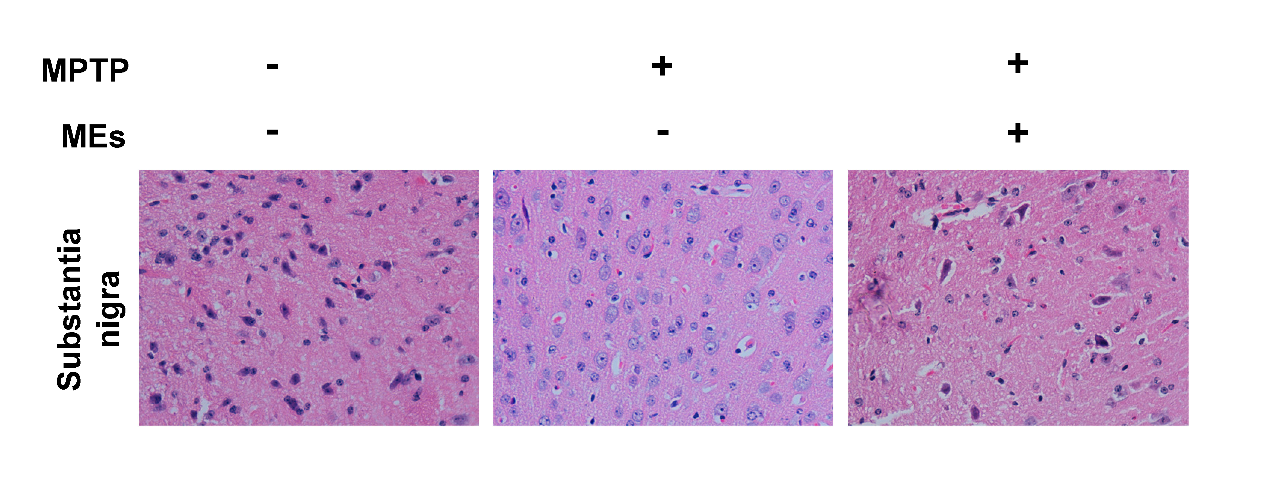
**Figure S4.** **MEs treatment increased neurons in the substantia nigra of MPTP-treated mice.** C57BL/6 mice (male 7-8 weeks old) were injected MPTP intraperitoneally at 30 mg/kg/day for five days, and MEs (30 mg/kg) were injected intraperitoneally for 5 days since the administration of MPTP. On day 7, mice were sacrificed and substantia nigra tissues were collected and hematoxylin and eosin (H&E) staining was performed. Scale bar =100 µm, right part (40×magnification).
